# Acute and persistent symptoms in non-hospitalized PCR-confirmed COVID-19 patients

**DOI:** 10.1038/s41598-021-92045-x

**Published:** 2021-06-23

**Authors:** Sofie Bliddal, Karina Banasik, Ole Birger Pedersen, Janna Nissen, Lisa Cantwell, Michael Schwinn, Morten Tulstrup, David Westergaard, Henrik Ullum, Søren Brunak, Niels Tommerup, Bjarke Feenstra, Frank Geller, Sisse Rye Ostrowski, Kirsten Grønbæk, Claus Henrik Nielsen, Susanne Dam Nielsen, Ulla Feldt-Rasmussen

**Affiliations:** 1 Department of Medical Endocrinology and Metabolism, Copenhagen University Hospital (Rigshospitalet), Copenhagen, Denmark; 2 Institute for Inflammation Research, Center for Rheumatology and Spine Diseases, Copenhagen University Hospital (Rigshospitalet), Copenhagen, Denmark; 3 Novo Nordisk Foundation Center for Protein Research, Faculty of Health and Medical Sciences, University of Copenhagen, Copenhagen, Denmark; 4 Department of Clinical Immunology, Zealand University Hospital, Køge, Denmark; 5 Department of Clinical Immunology, Copenhagen University Hospital (Rigshospitalet), Copenhagen, Denmark; 6 Department of Hematology, Rigshospitalet, Copenhagen, Denmark; 7 Biotech Research and Innovation Centre, Faculty of Health and Medical Sciences, University of Copenhagen, Copenhagen, Denmark; 8 Recurrent Pregnancy Loss Unit, Department of Fertility, Copenhagen University Hospital, Rigshospitalet, Fertility Clinic 4071, 2100 Copenhagen Ø, Denmark; 9 Recurrent Pregnancy Loss Unit, Department of Obstetrics and Gynecology, Hvidovre Hospital, 2650 Hvidovre, Denmark; 10 Methods and Analysis, Statistics Denmark, 2100 Copenhagen Ø, Denmark; 11 Management Section, Statens Serum Institut, Copenhagen, Denmark; 12 Department of Clinical Medicine, Faculty of Health and Clinical Sciences, Copenhagen University, Copenhagen, Denmark; 13 Department of Cellular and Molecular Medicine, University of Copenhagen, Copenhagen, Denmark; 14 Department of Epidemiology Research, Statens Serum Institut, Copenhagen, Denmark; 15 Department of Infectious Diseases, Copenhagen University Hospital (Rigshospitalet), Copenhagen, Denmark

**Keywords:** Infectious diseases, Viral infection, Health policy, Public health, Risk factors, Signs and symptoms, Comorbidities, Fatigue, Epidemiology, Outcomes research

## Abstract

Reports of persistent symptoms after hospitalization with COVID-19 have raised concern of a “long COVID” syndrome. This study aimed at determining the prevalence of and risk factors for acute and persistent symptoms in non-hospitalized patients with polymerase chain reaction (PCR) confirmed COVID-19. We conducted a cohort study of non-hospitalized participants identified via the Danish Civil Registration System with a SARS-CoV-2-positive PCR-test and available biobank samples. Participants received a digital questionnaire on demographics and COVID-19-related symptoms. Persistent symptoms: symptoms > 4 weeks (in sensitivity analyses > 12 weeks). We included 445 participants, of whom 34% were asymptomatic. Most common acute symptoms were fatigue, headache, and sneezing, while fatigue and reduced smell and taste were most severe. Persistent symptoms, most commonly fatigue and memory and concentration difficulties, were reported by 36% of 198 symptomatic participants with follow-up > 4 weeks. Risk factors for persistent symptoms included female sex (women 44% vs. men 24%, odds ratio 2.7, 95% CI 1.4–5.1, p = 0.003) and BMI (odds ratio 1.1, 95% CI 1.0–1.2, p = 0.001). In conclusion, among non-hospitalized PCR-confirmed COVID-19 patients one third were asymptomatic while one third of symptomatic participants had persistent symptoms illustrating the heterogeneity of disease presentation. These findings should be considered in health care planning and policy making related to COVID-19.

## Introduction

Coronavirus disease 2019 (COVID-19), caused by severe acute respiratory syndrome coronavirus 2 (SARS-CoV-2), has by May 2021 affected more than 158 million people worldwide and has been associated with 3.3 million deaths leading to a global health and financial crisis^1^. Established risk factors related to dying with the disease include high age, male sex and preexisting comorbidities^2–4^. A meta-analysis of 24,410 COVID-19 patients found that the most common symptoms were fever, cough, fatigue, and hyposmia^5^. However, not all who are infected develop symptoms^6,7^. Thus, there is a knowledge gap of individual resilience and risk factors determining the disease trajectory.

In the wake of the initial phase of the COVID-19 pandemic, several observational studies, patient groups and case series have reported persistent symptoms including reduced respiratory capacity^8^, fatigue and hyposmia^9–11^. In a follow-up study of 143 Italians discharged after hospitalization due to COVID-19, 87.4% still experienced COVID-19-related symptoms at 2 months after symptom start, and more than half of the patients reported persistent fatigue^12^. Similarly, Jacobs et al. found that fatigue was the most prevalent symptom 35 days post-hospitalization in 183 participants from the United States, and the majority of participants reported reduced quality of life and both physical and mental health problems^13^. A recent Chinese study presented 6-month follow-up data from 1733 hospitalized patients, of whom 63% still experienced fatigue and muscle weakness^14^. Clinical and public health interests are therefore no longer limited to information on mortality and clinical outcomes in hospitalized patients, but also to recovery and long-term consequences of COVID-19 post-hospitalization. Among other initiatives, this has led to the Post-Hospitalization COVID-19 study (PHOSP-COVID) in the United Kingdom aiming at long-term follow-up of 10,000 patients discharged after hospitalization due to COVID-19^15^. The increasing awareness of persistent symptoms among COVID-19 patients has even led to the designation “long COVID” which is yet to be clearly defined^16–18^. A recent first draft guideline on long-term effects of COVID-19 published by the National Institute of Health and Care Excellence (NICE) suggests to use the definition “ongoing symptomatic COVID-19” for symptoms lasting between 4 and 12 weeks after the acute onset and “post-COVID-19-syndrome” for symptoms lasting more than 12 weeks^19^.

Most studies on COVID-19 symptoms have recruited participants among hospitalized patients or patients attending outpatient clinics or specialized units, or support groups dealing with the consequences of COVID-19^5,9,20,21^. This confers a selection bias likely overestimating the true prevalence of symptoms and symptom severity. Thus, there is an academic knowledge gap in symptomatology among unselected non-hospitalized patients.

The aim of the present study was to determine the prevalence of and risk factors for acute and persistent symptoms in non-hospitalized patients with COVID-19 confirmed by a positive SARS-CoV-2 PCR.

## Materials and methods

### Participants

All individuals registered in the Danish Civil Registration System with a COVID-19 diagnosis confirmed by polymerase chain reaction (PCR) for SARS-CoV-2 by August 12th 2020 and an available biobank sample for genetic analyses were invited via the national digital postbox, e-Boks, to participate in a study on genetics in COVID-19 and related outcomes. The e-Boks is a secure digital postbox used by 92.1% of the Danish adult population by the second quarter of 2020^22^. A list of SARS-CoV-2 positive individuals was obtained from the Danish Microbiology (MiBa) database, which holds data for all SARS-CoV-2 PCR-tests in Denmark, provided by the Danish Health Data Authority^23,24^. Relevant biobank samples with sufficient material for genetic analyses were identified among stored samples in the Danish National Biobank or the Copenhagen Hospital Biobank^25,26^.

Invitations were sent to participants via e-Boks between June 24th and August 15th 2020 together with written project information and a link to a questionnaire on demographic data and symptomatology related to COVID-19. Interested participants could contact a call center between July 27th and August 28th 2020 to receive oral information on the project and provide oral consent prior to receiving a digital link for written consent. The last questionnaire was received digitally by October 31st 2020.

### Inclusion criteria

All individuals in the Danish Civil Registration System with a PCR-confirmed positive test for SARS-CoV-2 and an available biobank sample for genetic analyses with no history of hospitalization due to COVID-19 were eligible for this study. Hospitalization due to COVID-19 was defined as a hospital admission lasting longer than 12 h within 14 days of a positive PCR-test for SARS-CoV-2. Date of COVID-19 diagnosis and hospitalization status were accessed through the national register for COVID-19 surveillances (EpiMiBa). Non-hospitalized participants with a valid written consent and a completed questionnaire were included in this study.

### Questionnaire

All participants received a link in their e-Boks to a questionnaire with a large number of questions on demographic data as well as history and symptoms related to COVID-19 (median time to completion 13 min, interquartile range 10–18 min). The questionnaire was to a large extent based on a previously used questionnaire from the Danish Blood Donor Study^27^ and items on COVID-19 behaviour and symptoms were based on a questionnaire from Statens Serum Institut used for population monitoring at the beginning of the pandemic (“Influmeter”)^28^. The complete questionnaire is provided in Supplementary Appendix 1. Questions on lifestyle factors, demographics and self-reported co-morbidities included among other things sex (man or woman), smoking status (‘never’, ‘yes, sometimes’, ‘yes, daily, less than 10 times a day’, ‘yes, daily, 10 or more times a day’, ‘do not wish to answer’), height and weight, occupation in health care sector (‘no’, ‘yes, but I don’t have any patient contact’, ‘yes, I work with patients’), and the possibility to select the following chronic diseases (using layman terms): asthma, allergy (other than asthma), diabetes mellitus, hyperthyroidism, hypothyroidism, high blood pressure, heart attack, chest pain (angina pectoris), stroke, chronic bronchitis or big lungs or smokers lungs (emphysema, COPD), osteoarthritis, rheumatoid arthritis, osteoporosis, cancer, other.

Queries on symptomatology related to COVID-19 included the questions “Have you had a feeling of being ill in the period since February 1st 2020?” and if “yes” the following symptoms were listed with answer options of “No”, “Yes, a little”, “Yes, some”, “Yes, a lot”, “Don’t know”: Fever, chills, runny or stuffy nose, reduced sense of smell, reduced sense of taste, sneezing, sore throat, coughing, shortness of breath, headache, muscle and joint pain, chest pain, tiredness and exhaustion [fatigue], difficulties concentrating or remembering, lack of appetite, coloured sputum, red runny eyes, nausea, vomiting, diarrhoea, stomach pain, other. To clarify persistence of symptoms, the questionnaire included the question “Do you still have symptoms” and if the participant answered “yes”, the same list of symptoms was provided albeit without the option of ranking the degree of symptoms.

Symptoms commencing between 28 days before the positive SARS-CoV-2 PCR-test result and 14 days after the test result were included as COVID-19-related symptoms (Supplementary Fig. S1 online). Persistent symptoms were defined as symptoms lasting more than 4 weeks; these could be ongoing symptoms at the time of filling in the questionnaire in case of a follow-up time of more than 4 weeks from symptom onset. Alternatively, a stop date could be given in case of a reported symptom lasting more than 4 weeks from symptom onset to symptom stop. In sensitivity analyses, persistent symptoms lasting more than 12 weeks were explored based on the same selection criteria (12 weeks instead of 4 weeks). These time limits were in accordance with the time frame for ongoing symptomatic COVID-19 and post-COVID-19-syndrome, respectively, suggested by the National Institute for Health and Care Excellence in the United Kingdom^19^.

### Statistics

Demographic data were presented as appropriate by means and standard deviations (SD) or medians and inter-quartile range (IQR). Logistic regression analyses included as covariates: sex (male–female), age (calculated based on the Civil Registration number and date of data extraction), BMI, smoking status (non-smoker vs. ever smoker based on a composite score of confirmatory smoking categories), comorbidity (yes–no based on composite scores of self-reported chronic disease), work in the health care sector (yes–no), and time to questionnaire (weeks). The latter was included to account for any potential recall bias as the time periods between the PCR test and the questionnaire (in analyses of asymptomatic vs. symptomatic participants) or between the symptom start and the questionnaire (in analyses of persistent vs. no persistent symptoms among symptomatic individuals). Among the health care workers, almost all reported having patient contact [not specified if COVID-related] and thus patient contact was not included in the model. In the logistic regression analyses of risk of acute and persistent symptoms, we used a composite score (any symptom vs no symptom) across all acute and persistent symptoms, respectively. The composite score for acute symptoms included any symptom, regardless of reported severity. Participants answering “Don’t know” to the question of whether they still experienced symptoms were excluded from analyses regarding persistent symptoms (n = 13 at 4 weeks and n = 9 at 12 weeks follow-up). p-values are based on Welch Two Sample t-test for normally distributed traits and Fisher’s Exact test for categorical traits. All statistical analyses and preparation of figures were performed by R version 3.5.0 (the R foundation)^29^.

### Ethics

The study was part of the “Epidemiology and Genetics of COVID-19” study approved by the National Committee on Health Research Ethics (ID: NVK 2003947) and by the Data Protection Agency (ID: P-2020-356). All participants provided oral and written consent to participate.

## Results

**Figure 1:**
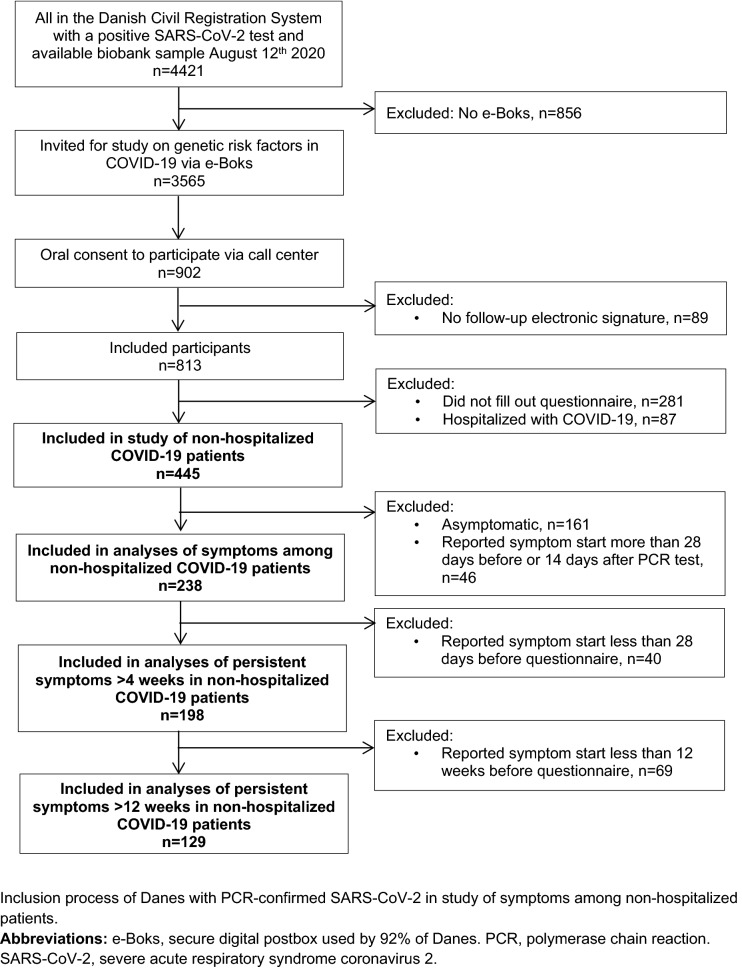
Flowchart of inclusion process. Inclusion process of Danes with PCR-confirmed SARS-CoV-2 in study of symptoms among non-hospitalized patients. *e-Boks* secure digital postbox used by 92% of Danes, *PCR* polymerase chain reaction, *SARS-CoV-2* severe acute respiratory syndrome coronavirus 2.

A total of 445 Danish non-hospitalized COVID-19 participants were included (see Fig. 1 for flow chart of the inclusion process). Among the invited participants (n = 3565), responders were slightly older and more often women compared to non-responders (median age 50 vs. 42 years, female:male 1.3 vs. 1.0). Most participants had been tested during the Spring of 2020 (Table 1). More women than men participated in the study and significantly more women worked in the health care sector and had patient contact (not specified if COVID-related). Also, the median date for PCR-testing of the included women was 1 month earlier than that of men (Table 1). There were no other significant differences in demographic data between men and women (Table 1). Less than half of the participants reported preexisting comorbidities; most commonly allergy (28%), hypertension (15%), osteoarthritis (17%), and asthma (8%) (see Supplementary Fig. S2 online).

**Table 1:** Characteristics of participants.

	Women (n = 254)	Men (n = 191)	p-value
Age (mean, SD)	45.6 (16.1)	48.7 (16.7)	0.04
BMI (median, IQR)	24.7 (6.9)	25.9 (4.6)	0.42
Smoking (ever, %)	14.2	16.2	0.59
Comorbidity (%)	55.5	56.3	1.00
Work in health care sector (%)	37.8	7.9	< 0.001
Work with patient contact (%)	36.2	7.3	< 0.001
Date of positive SARS-CoV-2 test (median, range)	22 May 2020 (8 Mar 2020–12 Aug 2020)	21 Jun 2020 (5 Mar 2020–12 Aug 2020)	0.001

Characteristics of included men and women with a history of COVID-19 confirmed by a SARS-CoV-2-positive PCR and without need of hospitalization within 14 days of the positive PCR-test for SARS-CoV-2.

*BMI* body mass index, *IQR* interquartile range, *PCR* polymerase chain reaction, *SARS-CoV-2* severe acute respiratory syndrome coronavirus 2, *SD* standard deviation.

### Acute symptoms

Completely asymptomatic COVID-19 was reported by 34% of participants. Asymptomatic participants had more often been tested towards the end of the study inclusion period (weeks from PCR to questionnaire; adjusted odds ratio (OR) 1.06 95% CI 1.02–1.09, p = 0.0008) (depicted in Supplementary Fig. S3 online), and were slightly older (OR for symptoms 0.98 (0.97–0.99), p = 0.004). There were no significant differences in sex, smoking status, comorbidities, health care worker status or BMI between those reporting symptoms and those without symptoms (p > 0.05).

**Figure 2:**
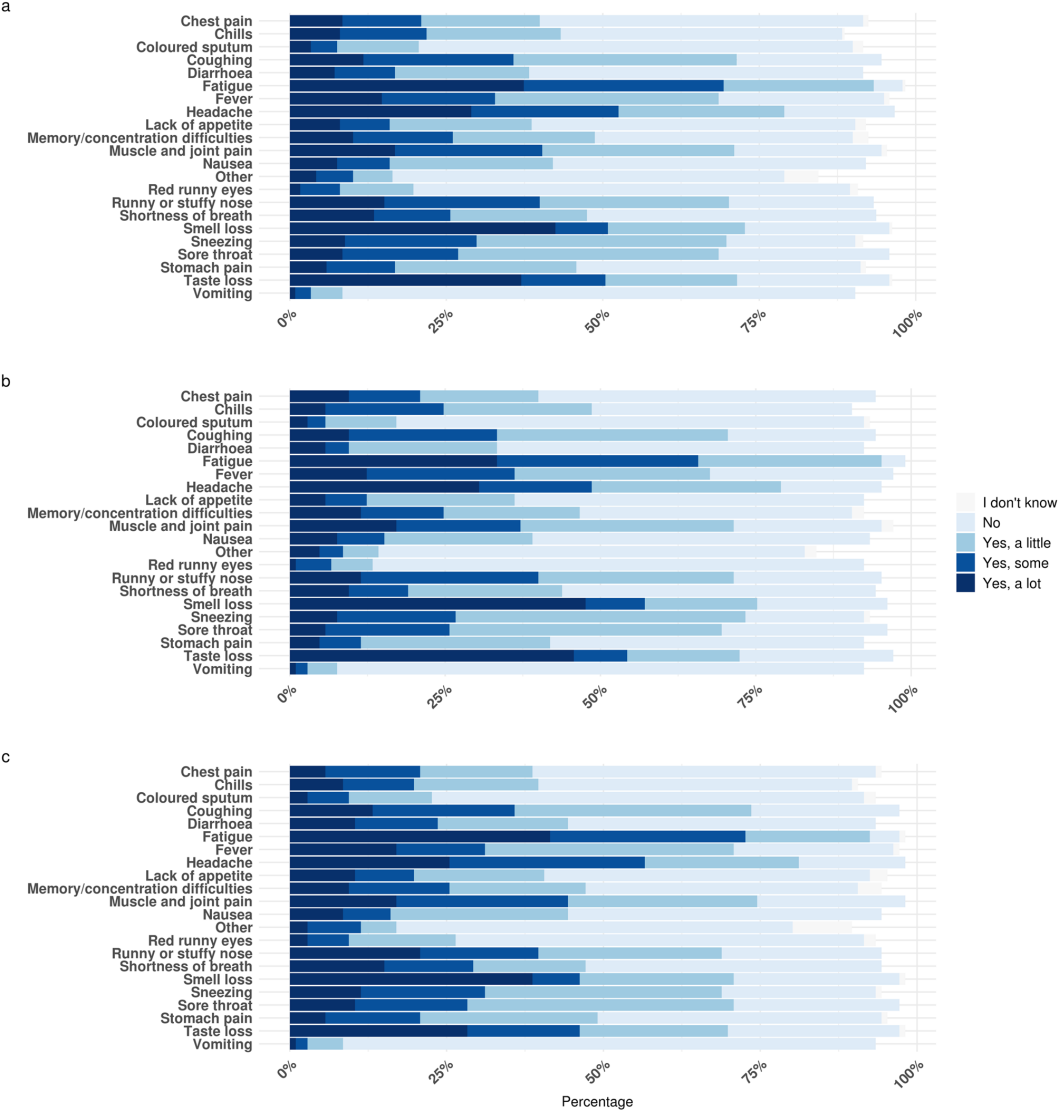
Symptom presentation and severity during acute COVID-19 in non-hospitalized patients. Symptom pattern and severity reported in relation to the acute phase of COVID-19 by non-hospitalized participants with a SARS-CoV-2-positive PCR test. Only participants with symptom start between 28 days before and 14 days after the PCR-test were included. (**a**) All symptomatic participants (n = 238). (**b**) Symptomatic participants without comorbidities (n = 105), and (**c**) symptomatic participants with self-reported comorbidities (n = 106) Twenty seven participants did not answer the questions on comorbidity. *PCR* polymerase chain reaction, *SARS-CoV-2* severe acute respiratory syndrome coronavirus 2.

Among the 238 participants experiencing symptoms, 55% of women and 52% of men had a sudden onset of symptoms arising within a few hours. Characteristics of symptomatic participants are presented in Table 2. Significantly more symptomatic women than men were working in the health care system, had contact with patients in their work and had been tested slightly earlier in the year (Table 2). The most common symptoms in the acute phase of COVID-19 were fatigue (95%), headache (82%), and sneezing (76%) (Fig. 2A). However, the symptoms most often reported as being severe were reduced smell, fatigue, and reduced taste (Fig. 2A). Excluding all participants with comorbidities did not change the symptom presentation (Fig. 2B).

**Table 2:** Characteristics of non-hospitalized symptomatic men and women with COVID-19.

	Women (n = 148)	Men (n = 90)	p-value
Age (mean, SD)	44.0 (15.9)	46.0 (14.8)	0.32
BMI (median, IQR)	24.3 (6.9)	25.5 (4.7)	0.55
Smoking (ever, %)	19.4	15.6	1
Comorbidity (%)	56.0	55.6	1
Work in health care sector (%)	44.0	13.3	< 0.001
Work with patient contact (%)	42.6	12.2	< 0.001
Contact to someone with positive test for COVID-19 (%)	49.4	49.1	1
Symptom start within a few hours (%)	55.4	52.2	0.24
Date of positive SARS-CoV-2 test (median, range)	12-May-2020 (10-Mar-2020–12-Aug-2020)	11-Jun-2020 (5-Mar-2020–12-Aug-2020)	0.14
Date of symptom onset (median, range)	7-May-2020 (2-Mar-2020–11-Aug-2020)	11-Jun-2020 (26-Feb-2020–13-Aug-2020)	0.09
Time from symptom start to PCR confirmation, days (median, range)	− 2 (− 23 to 6)	− 1 (− 17 to 6)	0.67
Time from symptom start to questionnaire, days (median, range)	104 (5–202)	69 (7–176)	0.01
Time from symptom start to stop, days (median, range) (n = 137)	14 (1–112)	12 (1–138)	0.06

Characteristics of 238 non-hospitalized symptomatic men and women with a history of COVID-19 confirmed by a SARS-CoV-2-positive PCR-test.

*BMI* body mass index, *IQR* interquartile range, *PCR* polymerase chain reaction, *SARS-CoV-2* severe acute respiratory syndrome coronavirus 2, *SD* standard deviation.

### Persistent symptoms

**Figure 3:**
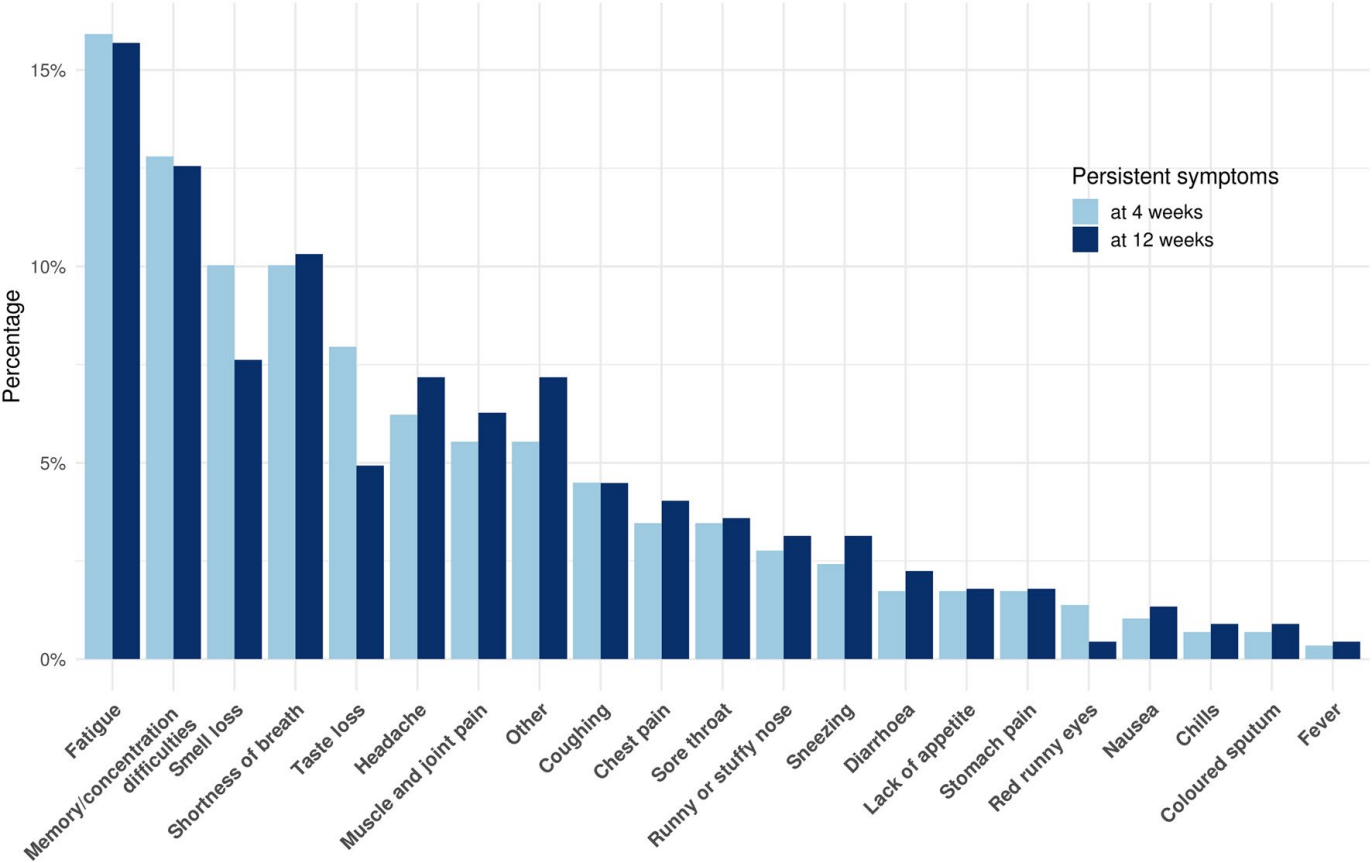
Persistent symptoms in COVID-19 at 4 and 12 weeks. Persistent symptoms after COVID-19. In dark blue, persistent symptoms for more than 4 weeks in the 198 non-hospitalized participants with symptoms and a follow-up time of more than 4 weeks and, in light blue, persistent symptoms for more than 12 weeks in the 129 non-hospitalized participants with symptoms and a follow-up time of more than 12 weeks.

Most symptomatic participants experienced symptom cessation within 2 weeks (n = 137, women in median 14 days (range 1–112), men in median 12 days (1–138)). The median time from symptom start to time of filling in the questionnaire was 90 days (range 5–197). Follow-up for more than 4 weeks was available from 198 symptomatic participants among whom persistent symptoms were reported by 36% (44% of women and 24% of men). Among the 198 participants, fatigue (16%), concentration or memory difficulties (13%), reduced sense of smell (10%), and shortness of breath (10%) were the most common persistent symptoms (Fig. 3). Among participants with more than 12 weeks of follow-up since symptom start (n = 129), 40% (48% of women and 23% of men) still had symptoms, especially fatigue (16%) and concentration difficulties (13%), following the same pattern as those with a shorter follow-up period (Fig. 3).

The risk of persistent symptoms more than 4 weeks after symptom start was significantly increased in women compared to men (OR 2.7 95% CI 1.4–5.2, p = 0.003 (Table 3)). Furthermore, there was an increased risk of persistent symptoms with increasing BMI (OR 1.1 95% CI 1.0–1.2, p = 0.001 (Table 3)). No other covariates were significantly associated with the risk of persistent symptoms. Female sex and BMI were also the only significant risk factors for persistent symptoms in the 129 participants with a follow-up period of more than 12 weeks (see Supplementary Table S1 available online). Supplementary Fig. S4 depicts the self-reported use of medication in the acute phase among the 198 symptomatic participants with 4 weeks of follow-up.

**Table 3:** Risk of persistent symptoms after COVID-19.

	Persistent symptoms (n = 72)	No persistent symptoms (n = 113)	Odds ratio (95% CI)	p-value	Adjusted odds ratio (95% CI)^a^	p-value
Sex^b^ (women, %)	76.4%	54.9%	2.66 (1.38–5.14)	0.003	2.91 (1.32–6.39)	0.008
Age (mean (SD))	47.4 (15.2)	45.4 (15.5)	1.01 (0.99–1.03)	0.38	1.00 (0.97–1.03)	0.97
Smoking (ever, %)^c^	16.7%	15.0%	1.13 (0.50–2.53)	0.84	0.84 (0.30–2.41)	0.75
BMI (median (IQR))	26.8 (8.6)	24.4 (4.3)	1.11 (1.04–1.18)	0.001	1.13 (1.05–1.22)	0.001
Comorbidity (%)	47.2%	42.3%	1.47 (0.77–2.80)	0.26	1.40 (0.68–2.85)	0.36
Work in health care sector (%)	43.1%	29.5%	1.81 (0.98–3.36)	0.08	1.28 (0.58–2.81)	0.54
Time from symptom start to questionnaire (weeks) (median, range)	16 (5–25)	15 (5–27)	1.01 (0.96–1.06)	0.76	0.99 (0.93–1.06)	0.86

*BMI* body mass index, *CI* confidence interval, *IQR* interquartile range.

^a^The risk of persistent symptoms vs. no persistent symptoms after 4 weeks in participants with symptoms in the acute phase and a minimum of 4 weeks of follow-up from symptom start (n = 198) adjusted for: sex, age, smoking, BMI, comorbidity and time from symptom start to questionnaire. Participants answering “I don’t know” to whether they still experienced symptoms at follow-up were excluded from this analysis (n = 13).

^b^Men as reference group.

^c^Non-smokers as reference group.

More women than men worked in the health care sector (Tables 1 and 2). Thus, we re-ran analyses of risk factors for persistent symptoms by only including the 117 women with follow-up of more than 4 weeks. Doing so, BMI was still a significant risk factor (adjusted OR 1.2 95% CI 1.1–1.3, p = 0.003), but being a health care worker was not (OR 1.5 95% CI 0.6–3.8, p = 0.39).

**Figure 4:**
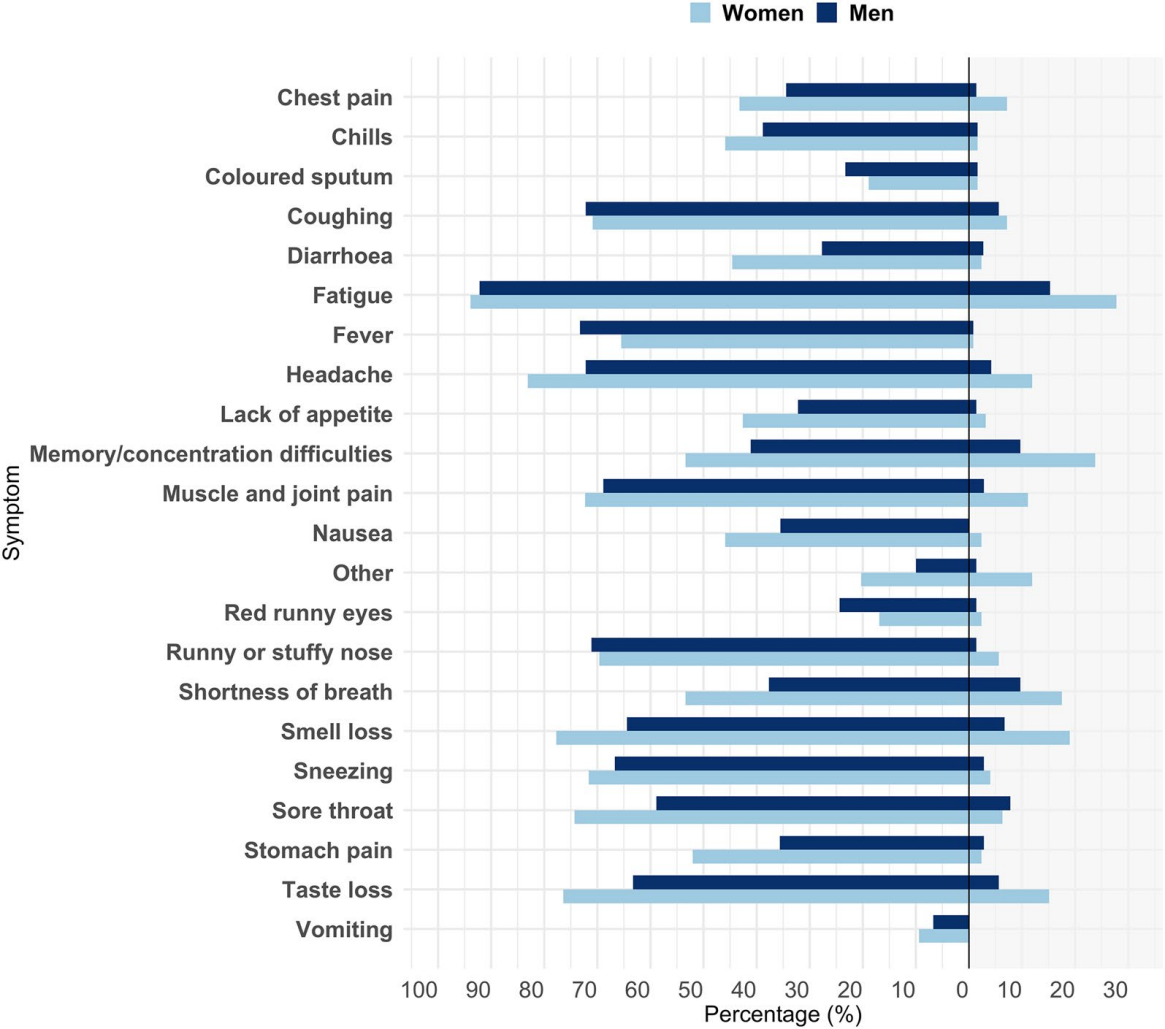
Acute symptoms and symptoms > 4 weeks in non-hospitalized men and women with COVID-19. The percentage of acute (panel to the left) symptoms and symptoms > 4 weeks (panel to the right) in non-hospitalized COVID-19 participants according to sex. Acute symptoms were reported by 148 women and 90 men of which 4 weeks of follow-up was available in 126 women and 72.

Furthermore, we depicted acute and persistent symptoms according to sex (Fig. 4). Men and women had a similar prevalence of fatigue in the acute phase (Fig. 4 left hand side). In participants with persistent symptoms, fatigue was the most common persistent symptom in both men and women, but the prevalence of persistent fatigue was almost twice as high in women compared to men (28% vs. 15%, OR 2.2 95% CI 1.0–4.7, p = 0.05, right side of Fig. 4).

## Discussion

The present study showed that among non-hospitalized PCR-confirmed COVID-19 patients, one third were asymptomatic while one third of symptomatic participants had persistent symptoms after 4 and 12 weeks. The most common persistent symptoms were fatigue and memory and concentration difficulties. The risk of persistent symptoms was higher in women and in participants with a higher BMI.

Unlike most previous studies on COVID-19-related symptoms an important strength of the present study was the recruitment of non-hospitalized participants with a positive SARS-CoV-2 PCR-test. Thus, we were able to depict variations in symptoms even among non-hospitalized participants with a confirmed COVID-19 diagnosis. Contrary to studies relying solely on information gathered through questionnaire data, our use of national register data on SARS-CoV-2 PCR tests and hospitalization strengthens the validity of the study results. However, an important limitation to our study was the quite low response rate to the study invitations (approximately 20%) with responders being slightly older and more often women. Potential participants suffering from more severe fatigue or difficulties concentrating could be less likely to respond to our study invitation or be able to complete the questionnaire. Furthermore, our study finding of women being at higher risk of persistent symptoms may be biased by the fact that more asymptomatic individuals, especially men, were included towards the end of the inclusion period and were slightly older. This likely reflects both an altered test strategy in Denmark with easier access to targeted testing for mild cases of COVID-19 and screening of close contacts, and a survival bias with a higher risk of hospitalization among older citizens and symptomatic men. In addition, more women worked in the health care sector with patient contact and although this was not associated with an increased risk of persistent symptoms in the present study, larger studies are needed to clarify if the increased risk of persistent symptoms among women could be confounded by specific lines of work. Finally, all studies of COVID-19-related symptoms suffer from an inherent likelihood of attracting participants with a personal experience of severe or persistent symptoms thus risking an overestimation of the true prevalence of persistent symptoms. A similar selection bias may be present in our study, however, likely reduced compared to most studies specifically addressing COVID-19-related symptoms because we specifically invited participants to a study on genetics and not symptoms.

There are other limitations to the present study. First, the study was designed to only include SARS-CoV-2 positive participants with an available biobank sample for genetic analyses. The biobank samples in the Danish National Biobank mainly consisted of samples from the Danish Blood Donor Study (with a bias towards healthy participants) and from the Copenhagen Hospital Biobank using leftover material from hospitalized patients (with an inherent bias towards participants with comorbidity). However, we were able to depict our findings according to self-reported comorbidities and both acute and persistent symptoms were similar between the previously healthy participants and those with comorbidities. Second, although based on previous large population studies our questionnaire was not validated and only included the most common symptoms reported with COVID-19 at the time as opposed to the more than 200 symptoms identified by Davis et al.^30^. Third, the cross-sectional design and the fact that symptoms severity was only reported for symptoms in the acute phase hindered the possibility to detect changes in symptom intensity over time. Thus, long-term studies with longitudinal follow-up are needed to assess different trajectories in recovery.

Our finding of fatigue as the most prevalent persistent symptom matches previous studies among post-hospitalized COVID-19 patients suffering from fatigue^9,12,31,32^. To our knowledge, few studies have been published on persistent symptoms in non-hospitalized patients with positive SARS-CoV-2 PCR-tests. Among 292 participants interviewed by phone at a median of 16 days post-testing, Tenforde et al. found 35% of participants with acute symptoms to still be experiencing symptoms (especially coughing, fatigue and shortness of breath)^33^. In a study of 180 participants (eight of whom had been hospitalized) interviewed during the acute phase of disease and at follow-up phone calls, persistent symptoms were reported by 53% of symptomatic participants at a mean follow-up time of 125 days (most often fatigue, loss of smell and taste, and arthralgias)^34^. Larger survey-based studies on COVID-19-related symptoms in a multitude of participants with self-reported COVID-19 are available online, although not yet peer-reviewed^30,35–39^. Characteristic for most of these studies is the acceptance of self-reported COVID-19 status by participants without laboratory confirmation of COVID-19-status, and recruitment of participants with the specific aim of COVID-19 symptom reporting likely inferred a substantial selection bias. However, most studies do report fatigue, concentration problems and loss of smell and taste as the most common persistent symptoms, which is comparable to our findings in PCR-confirmed COVID-19 non-hospitalized participants^30,34–38^. Thus, our study provides substantial laboratory- and registry-confirmed evidence in support of these symptoms being part of a “long COVID”-syndrome existing even in non-hospitalized COVID-19 patients.

Why SARS-CoV-2 carries a high risk of persistent symptoms, especially in women, is an urgent research question. Suggested mechanisms fall into two categories of either direct effects from the virus or indirect effects caused by immune responses to the virus^40^. While persistent symptoms including chronic fatigue are well-described in other pulmonary infections such as SARS and pneumonia, SARS-CoV-2 may differ in its multi-organ-affection and high contagiousness^40–43^. The female preponderance bears resemblance to that of many autoimmune diseases^44^. Unfortunately, the present study did not have laboratory data to assess either initial or persistent immunological response against SARS-CoV-2 and did not have data for retesting for SARS-CoV-2 to ascertain resolution of the viral load in participants with persistent symptoms. However, previous studies have found a poor correlation between the risk of persistent symptoms and objective measures of disease despite performing a wide range of clinical and laboratory tests in COVID-19 patients either during the acute phase or at follow-up^9,31^. Also, a negative SARS-CoV-2 PCR-test as a sign of recovery has been criticized for not corresponding to the patients’ perception of (or lack of) recovery^30,37^. Thus, detailed laboratory data and functional studies are urgently needed to understand the immunological component of long COVID.

An important question arising from the present and other studies of long COVID is how to manage these patients with persistent symptoms. Previous studies of chronic fatigue have shown uncertain benefit of treatment such as exercise, cognitive behavioral therapy, and rehabilitation initiatives, and are, nevertheless, not necessarily compatible with post-COVID fatigue^45–47^. Several specific management initiatives are emerging for “long COVID” as well as guideline initiatives by the National Institute for Health and Care Excellence in United Kingdom, and guidance material for self-rehabilitation after COVID-19 released by the World Health Organization^9,12,19,48^. Some have argued for the importance of involving general practitioners in the management of this syndrome^49^ and some have advocated for the importance of including patients in emerging new strategies^16,50^. A subset of patients has yet to recover after months of symptoms and those patients may need long-term follow-up at specialized units. Thus, an important aspect of future studies on this matter is to identify patients at risk of long-term symptoms. The present study suggests that up to 40% of non-hospitalized previously healthy patients will experience some degree of persistent symptoms with an increased risk in women. Larger studies of big data including real-time symptom reporting will aid in determining risk factors and predicting disease trajectories^36,51^. The mounting evidence of “long COVID” calls for active policy making to secure prevention and early detection, facilitate optimal treatment and support rehabilitation^16,40^.

Equally important may be the high proportion of asymptomatic COVID-19 individuals. In the present study 34% of non-hospitalized PCR-confirmed SARS-CoV-2-positive participants reported no symptoms in the acute phase of the disease. However, the overall prevalence of asymptomatic COVID-19 was similar to that estimated in Danish health care workers (assessed by IgM and IgG antibodies against SARS-CoV-2) of 46.5%^52^. Similarly, a study of the Icelandic population found that 43% were asymptomatic at time of a positive SARS-CoV-2 PCR test among participants included in a population screening unlike targeted testing of severely symptomatic individuals^53^. Furthermore, the COVID Symptom Score study prospectively monitored more than 2 million citizens in UK, US and Sweden. In a subset of 26,495 participants with a self-reported positive SARS-CoV-2 PCR-test, 55.3% were asymptomatic^36,51^ [numbers deduced from “Supplementary files”]. Although some studies reported a lower prevalence^34^, there did seem to be a general agreement on a true high prevalence of asymptomatic COVID-19. In a recent analytical model based on the assumption that 30% of COVID-patients may by asymptomatic, Johansson et al. found that asymptomatic individuals would carry 50% of all transmissions^54^. This has important implications for disease control and stresses the need for active tracing and testing of close contacts as part of disease control.

In conclusion, even among non-hospitalized PCR-confirmed SARS-CoV-2-positive participants, more than one third reported persistent symptoms after 4 and 12 weeks, respectively. The most prevalent persistent symptoms were fatigue and memory and concentration difficulties. Contrary to this, 34% of non-hospitalized patients were asymptomatic in the acute phase of the disease stressing the importance of testing near contacts. The high prevalence of both patients with persistent symptoms and of asymptomatic patients bear witness to the heterogeneity of the disease presentation. Although data should be reproduced due to possible limitations of survival or recall bias, these findings should be taken into account in future health care planning and policy making related to COVID-19 prevention, detection, treatment and follow-up.

## Supplementary Information

Supplementary Information.

## Data Availability

The data that support the findings of this study are available from The Danish COVID-19 Genetic Consortium but restrictions apply to the availability of data used under license for the current study, and thus are not publicly available. Data are however available from the authors upon reasonable request and with permission from The Danish COVID-19 Genetic Consortium under Danish legislation.
