# Supplementary material for: Acute and persistent symptoms in non-hospitalized PCR-confirmed COVID-19 patients
Source: Sci Rep. 2021 Jun 23;11:13153. doi: 10.1038/s41598-021-92045-x (PMC8222239; doi:10.1038/s41598-021-92045-x)
Supplement: Supplementary file 1 — Supplementary Information. [file 41598_2021_92045_MOESM1_ESM.pdf]

Supplementary files

Acute and persistent symptoms in non-hospitalized PCR-confirmed COVID-19 patients

Sofie Bliddal¶[1,2], Karina Banasik¶[3], Ole Birger Pedersen[4], Janna Nissen[5], Lisa Cantwell[3], Michael Schwinn[5], Morten Tulstrup[6,7], David Westergaard[3,8,9], Henrik Ullum[10,11], Søren Brunak[3], Niels Tommerup[12], Bjarke Feenstra[13], Frank Geller[13], Sisse Rye Ostrowski[5,11], Kirsten Grønbæk[6,7,11], Claus Henrik Nielsen[2,11], Susanne Dam Nielsen[11,14], Ulla Feldt-Rasmussen[1,11]*, on behalf of The Danish COVID-19 Genetic Consortium^.

1. Department of Medical Endocrinology and Metabolism, Copenhagen University Hospital (Rigshospitalet), Denmark.
2. Institute for Inflammation Research, Center for Rheumatology and Spine Diseases, Copenhagen University Hospital (Rigshospitalet), Denmark.
3. Novo Nordisk Foundation Center for Protein Research, Faculty of Health and Medical Sciences, University of Copenhagen, Denmark.
4. Department of Clinical Immunology, Zealand University Hospital, Køge, Denmark
5. Department of Clinical Immunology, Copenhagen University Hospital (Rigshospitalet), Denmark
6. Department of Hematology, Rigshospitalet, Copenhagen, Denmark
7. Biotech Research and Innovation Centre, Faculty of Health and Medical Sciences, University of Copenhagen, Copenhagen, Denmark
8. Recurrent Pregnancy Loss Unit, Capital Region, Copenhagen University Hospital, Rigshospitalet, Fertility Clinic 4071, 2100 Copenhagen Ø, and Hvidovre Hospital, 2650 Hvidovre, Denmark
9. Methods and Analysis, Statistics Denmark, 2100 Copenhagen Ø, Denmark
10. Management section, Statens Serum Institut, Copenhagen, Denmark
11. Department of Clinical Medicine, Faculty of Health and Clinical Sciences, Copenhagen University, Denmark.
12. Department of Cellular and Molecular Medicine, University of Copenhagen, Copenhagen, Denmark
13. Department of Epidemiology Research, Statens Serum Institut, Copenhagen, Denmark
14. Department of Infectious Diseases, Copenhagen University Hospital (Rigshospitalet), Denmark.

List of supplementary files

Supplemental figure S1. Time between symptom start and positive SARS-CoV-2 PCR-test

Supplemental figure S2. Comorbidities reported by non-hospitalized COVID-19 patients

Supplemental figure S3. Prevalence of participants with acute symptoms and no acute symptoms according to date of PCR test

Supplemental figure S4. Medication used in the acute phase of symptoms according to presence of persistent symptoms

Supplementary table S1. Risk factors for persistent symptoms after 12 weeks

Appendix 1. Questionnaire used in the study – English translation

Appendix 2. Consortium

Supplemental figure S1. Time between symptom start and positive SARS-CoV-2 PCR-test

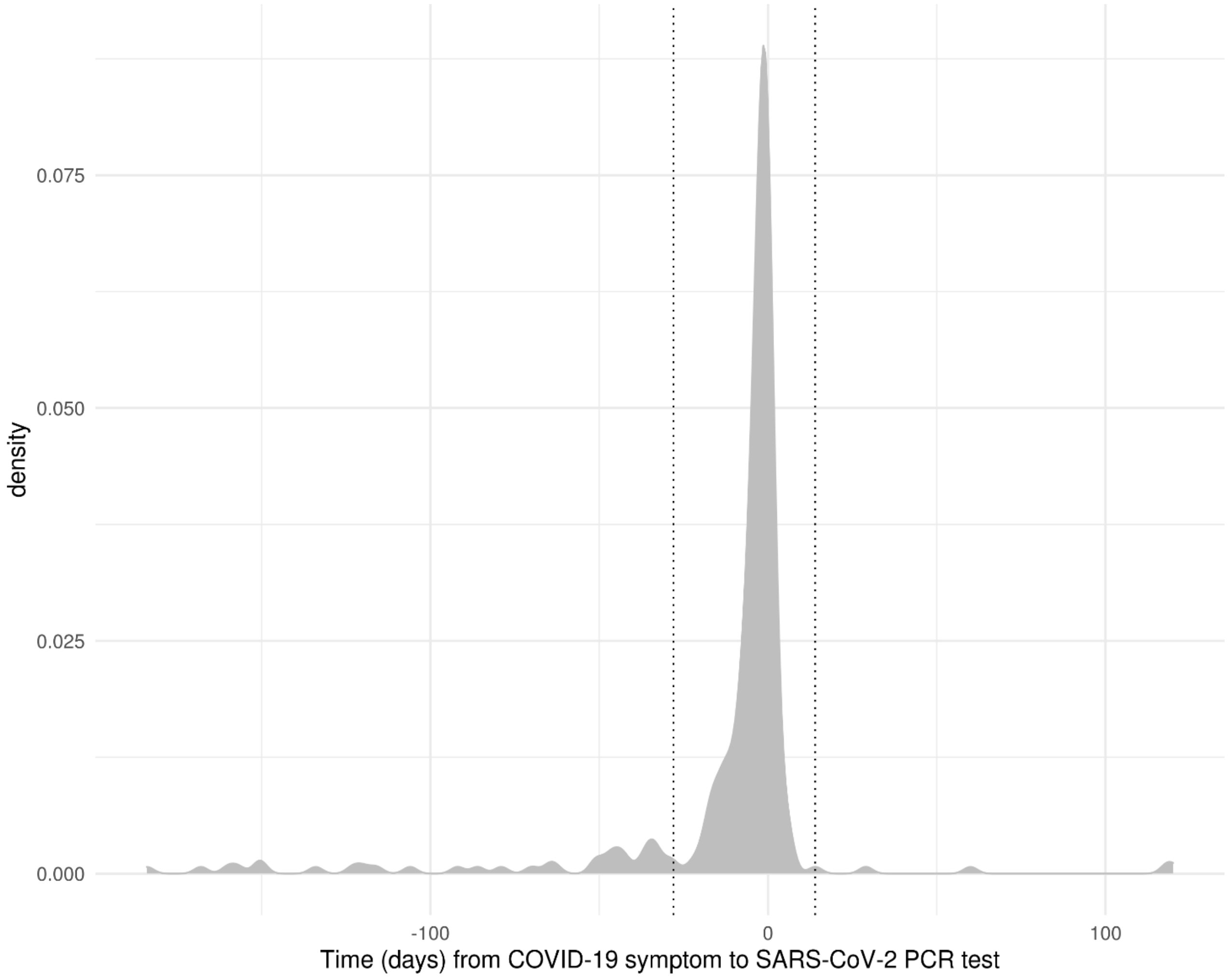

Time in days from symptom start to SARS-CoV-2 PCR test in 445 non-hospitalized participants with COVID-19. Participants with symptom start up to 28 days before and 14 days after the PCR test (vertical dotted lines) were considered to have COVID-related symptoms (n=235), while participants with no symptoms (n=161) or with symptoms outside of this time frame (n=46) were not included in analyses of COVID-19 symptoms.

Supplemental figure S2. Self-reported comorbidities in non-hospitalized COVID-19 patients

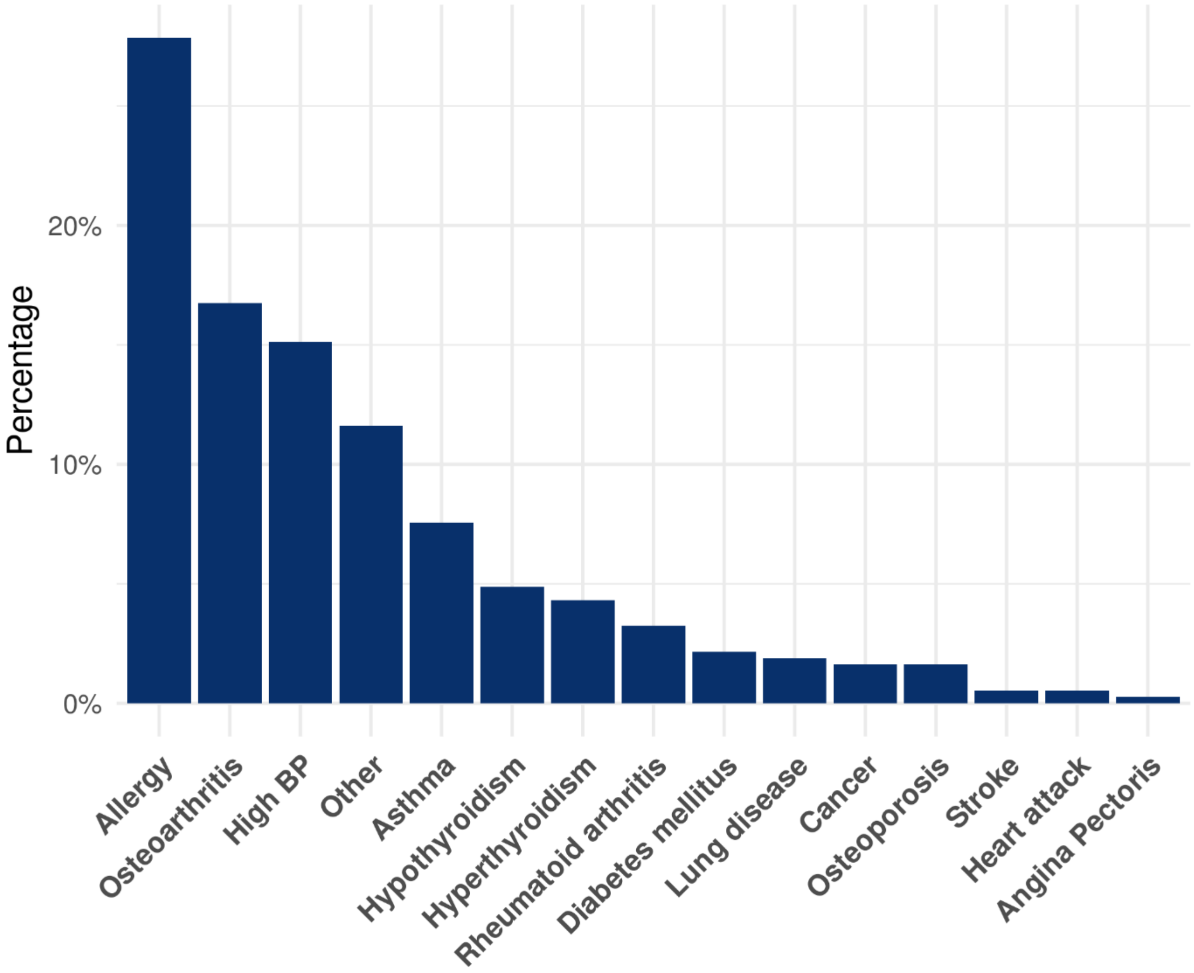

Self-reported comorbidities in 445 non-hospitalized participants with a history of COVID-19 confirmed by SARS-CoV-2-positive PCR test. A total of 193 (43.4%) participants reported of comorbidities.
Abbreviations: BP, blood pressure. PCR, polymerase chain reaction. SARS-CoV-2, severe acute respiratory syndrome coronavirus 2.

Supplemental figure S3. Prevalence of participants with acute symptoms and no acute symptoms according to date of PCR test

C

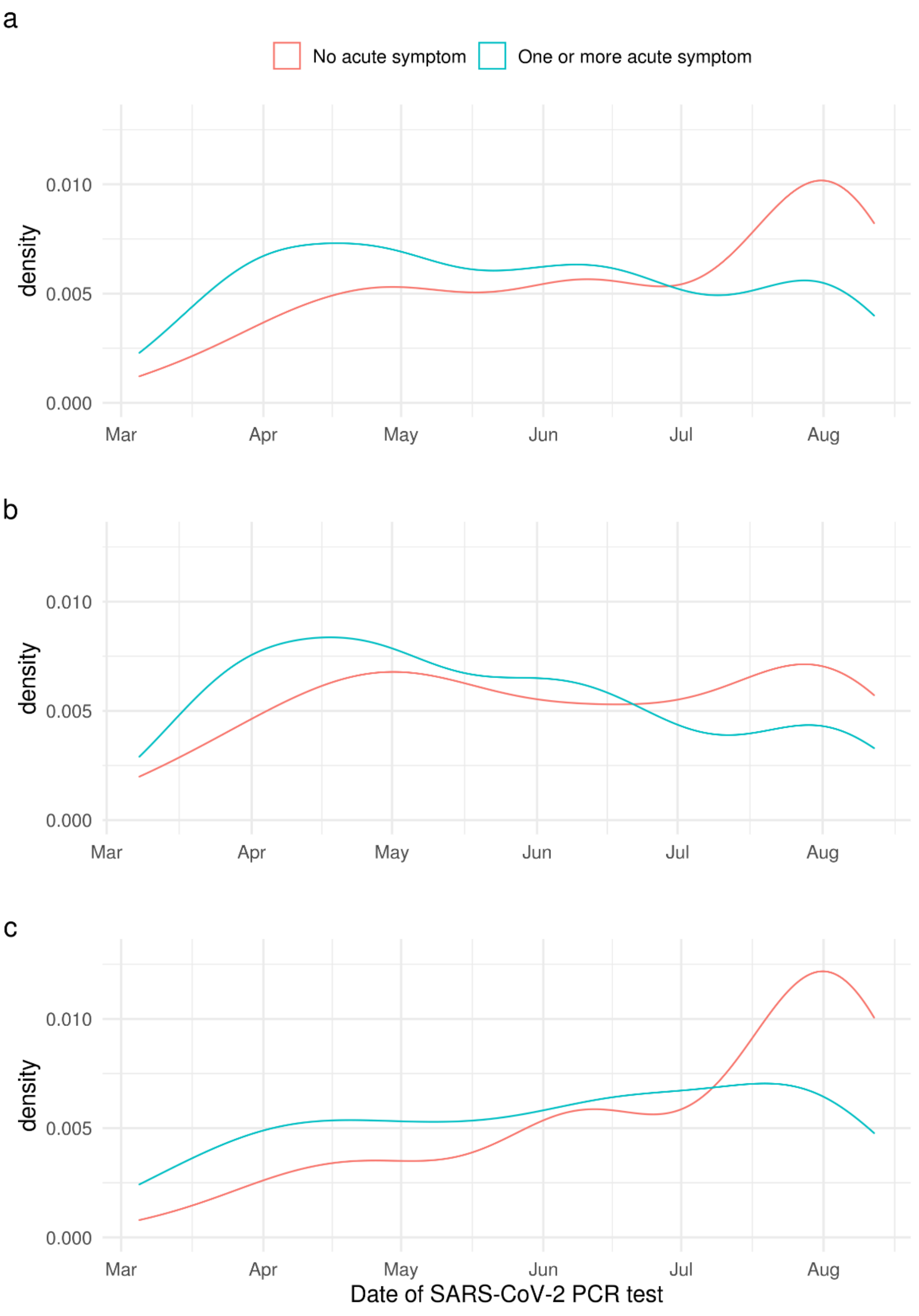

Prevalence of participants with (blue line) and without (red line) acute symptoms according to date of SARS-CoV-2 PCR-test. A, all participants. B, women only. C. Men only.

Supplemental figure S4. Medication used in the acute phase of symptoms according to presence of persistent symptoms

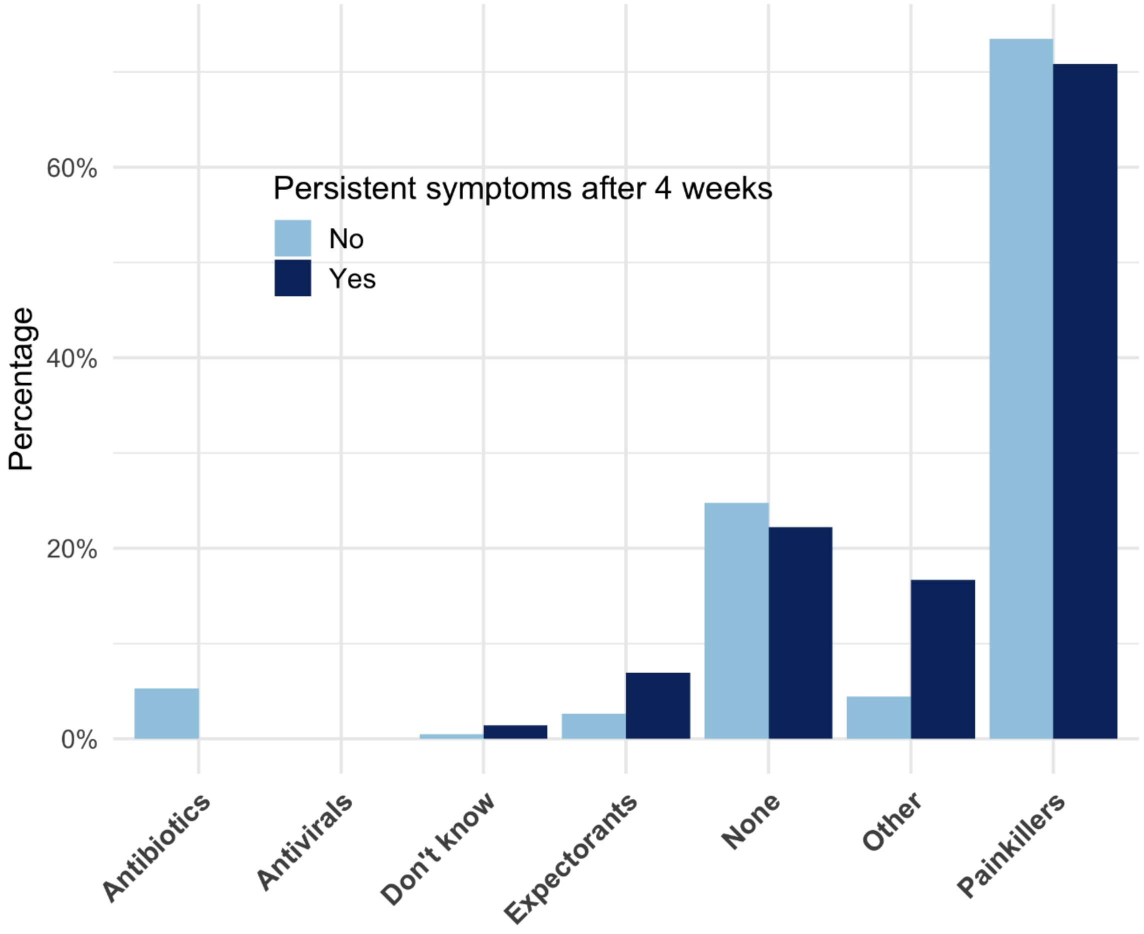

Use of medication in the acute phase among 198 participants experiencing symptoms in the acute phase and with a minimum of four weeks follow-up. All participants had a history of non-hospitalization during COVID-19 confirmed by SARS-CoV-2-positive PCR test.

Supplementary Table S1. Risk of persistent symptoms >12 weeks after COVID-19

	Persistent symptoms (n=52)	No persistent symptoms (n=68)	Odds ratio (95% CI)	p-value	Adjusted odds ratio (95% CI)*	p-value
**Sex** (women, %)**	82.6%	60.3%	3.15 (1.32-7.49)	0.009	6.01 (2.00-18.07)	0.001
**Age (mean (SD))**	45.0 (14.2)	44.7 (13.2)	1.00 (0.98-1.03)	0.90	1.00 (0.96-1.03)	0.84
**Smoking (ever, %)*** **	21.2%	16.2%	1.39 (0.55-3.51)	0.49	1.68 (0.44-6.46)	0.45
**BMI (median (IQR))**	27.0 (8.2)	24.4 (3.8)	1.12 (1.03-1.21)	0.01	1.18 (1.05-1.31)	0.004
**Comorbidity (%)**	48.1%	39.7%	1.75 (0.79-3.85)	0.23	2.45 (0.96-6.25)	0.06
**Time from symptom start to questionnaire (weeks) (median, range)**	16.9 (12.1-25.2)	19.9 (12.2-27.2)	0.89 (0.80-0.98)	0.02	0.88 (0.78-1.00)	0.06

*The risk of persistent symptoms vs. no persistent symptoms after twelve weeks in participants with symptoms in the acute phase and a minimum of four weeks of follow-up from symptom start (n=129) adjusted for: sex, age, smoking, BMI, comorbidity and time from symptom start to questionnaire. Participants answering “I don’t know” to whether they still experienced symptoms at follow-up were excluded from this analysis (n=9).
**Men as reference group.
***Non-smokers as reference group.
Abbreviations: BMI, body mass index. CI, confidence interval. IQR, interquartile range.4

Appendix 1. Questionnaire

COVIDGWASVI ENGLISH TRANSLATION

Because you have previously been tested for infection with SARS-CoV-2 commonly known as corona virus, we would like to ask you to answer a questionnaire on your health, how the corona virus pandemic has influenced your life, on infection with corona virus and the risk of spreading the disease.

We would like to ask you about your household, your employment, your transportation, your health, and symptoms on corona virus infection.

The answers will be used for research in the spread of the infection and into why some catch the disease and others do not. You should know that participation is voluntary.

If there are questions you do not wish to answer you can choose to skip those. The questionnaire takes approximately 20 minutes to complete.

Thank you for your help

The questionnaire contains 47 questions.

Preliminary questions

[]Are you a man or a woman? *

Choose one of the answers below:

- ○Woman
- ○Man

Symptoms

The following questions regard corona virus and risk factors

[]

How often do you normally catch a cold or experience flu-like symptoms?

Choose one of the answers below:

- ○Never

- 1-2 times a year
- Between 3 and 5 times a year
- Between 6 and 10 times a year
- More than 10 times a year
- I don’t know

[]

Have you been vaccinated against pneumonia (pneumococcal infection)?

Choose one of the answers below:

- Yes
- No
- I don’t know / don’t recall

[]

Have you been vaccinated against the flu this fall/winter 2019-2020?

Choose one of the answers below:

- Yes
- No
- I don’t know / don’t recall

[]Have you felt ill since February 1st 2020?

Choose one of the answers below:

- Yes
- No
- Don’t know

[]

Have you had any of the following symptoms since February 1st 2020? You can choose multiple statements

[Only answer this question if the following is true: Answer 'Yes' to question '5 [DCS5]' (Have you felt ill since February 1st 2020?)

Choose one answer to each element:

	No	Yes, a little	Yes, some	Yes, a lot	Don’t know
**Fever**	○	○	○	○	○

	No	Yes, a little	Yes, some	Yes, a lot	Don’t know
Chills	○	○	○	○	○
Runny or stuffy nose	○	○	○	○	○
Reduced sense of smell loss	○	○	○	○	○
Reduced sense of taste	○	○	○	○	○
Sneezing	○	○	○	○	○
Sore throat	○	○	○	○	○
Coughing	○	○	○	○	○
Shortness of breath	○	○	○	○	○
Headache	○	○	○	○	○
Muscle and joint pain	○	○	○	○	○
Chest pain	○	○	○	○	○
Tiredness and exhaustion	○	○	○	○	○
Difficulties concentrating or remembering	○	○	○	○	○
Lack of appetite	○	○	○	○	○
Coloured sputum	○	○	○	○	○
Red runny eyes	○	○	○	○	○
Nausea	○	○	○	○	○
Vomiting	○	○	○	○	○
Diarrhoéa	○	○	○	○	○
stomach pain	○	○	○	○	○
Other	○	○	○	○	○

COVID-19 risks

[]Approximately when did the symptoms start?

[Only answer this if the following is true: Answer 'Yes' to question '5 [DCS5]' (Have you felt ill since February 1st 2020?]

Enter a date:

[]Do you still have symptoms?

[Only answer this if the following is true: Answer 'Yes' to question '5 [DCS5]' (Have you felt ill since February 1st 2020?)]

Choose one of the answers below:

- ○Yes
- ○No
- ○Don’t know

[]

Which symptoms do you still have? You can choose multiple answers

Only answer this if the following is true:
Answer 'Yes' to question '8 [CDR21]' (Do you still have symptoms?)

Choose on or more answers:

- ☐Fever
- ☐ chills
- ☐ runny or stuffy nose
- ☐ reduced sense of smell
- ☐ reduced sense of taste
- ☐ sneezing
- ☐ sore throat
- ☐ coughing
- ☐ shortness of breath
- ☐ headache
- ☐ muscle and joint pain
- ☐ chest pain
- ☐ tiredness and exhaustion
- ☐ difficulties concentrating or remembering
- ☐ lack of appetite
- ☐ coloured sputum
- ☐ red runny eyes

- ☐ nausea
- ☐ vomiting
- ☐ diarrhoea
- ☐ stomach pain
- ☐Other

[]Approximately when did the symptoms stop?

[Only answer this if the following is true:
Answer 'No' to question '8 [CDR21]' (Do you still have symptoms?)]

Enter a date:

[]

[Only answer this if the following is true:
Answer 'Yes, some' *or* 'Yes, a lot' *or* 'Yes, a little' to question '6 [DCS6]' (Have you had any of the following symptoms since February 1st 2020?? You can choose multiple statements (Fever))]

Choose one answer for each element:

	Yes	No
**Did you measure your temperature, when you had a fever?**	○	○

[]What was the highest temperature you measured?

Only answer this if the following is true:
Answer 'Yes' to question '11 [DCR10]' (Did you take your temperature, when you had a fever?)

Write you answer here:

[]where did you measure your temperature?

Only answer this if the following is true:
Answer 'Yes' to question '11 [DCR10]' (Did you take your temperature, when you had a fever?)

Choose one of the answers below:

- ○Ear

- ○Forehead
- ○Oral
- ○Rectal
- ○Other

[]The following questions concern whether you have been at risk of being exposed to corona virus.

[Only answer this if the following is true:
Answer 'Yes' to question '5 [DCS5]' (Have you felt ill since February 1st 2020?)]

Choose one answer for each element below:

	Yes	No	Don’t know
**Did you travel to a corona virus high risk area within 14 days of your symptom start?**	○	○	○
**Within 14 days of your symptom start, were you a near contact to someone who had been in a corona virus high risk area?**	○	○	○
**Have you been a near contact to someone who tested positive for corona virus?**	○	○	○
**Did your symptoms arise suddenly (within few hours)?**	○	○	○

[]Where have you had contact with those whom you know have tested positive for corona virus (COVID-19)?

Only answer this if the following is true:
Answer 'Yes' to question '14 [DCR1]' (The following questions regards whether you have been at risk of corona virus transmission. (Have you been in near contact with someone whom you know has tested positive for corona virus?))

Choose one of the answers below:

- Household
- Family or friends outside of your household
- School or daycare
- Institution
- At work or education
- Other

[]Approximately when was the last time you were together with one or more of these before you fell ill?

Only answer this if the following is true:
Answer 'Yes' to question '14 [DCR1]' (The following questions regards whether you have been at risk of corona virus transmission. (Have you been in near contact with someone whom you know has tested positive for corona virus?))

Enter a date:

[]

Did you take any medication to relieve your symptoms? (Choose one or more answers)

[Only answer this if the following is true: Answer 'Yes' to question '5 [DCS5]' (Have you felt ill since February 1st 2020?]

Choose one or more answers:

- No, I did not take any medication
- Yes, pain killers (fx acetaminophen, ibuprofen, strepfen)
- Yes, antitussives or mucus-loosening (fx dexofan, flavamid, pectyl, mucolysin)
- Yes, antiviral medication (Tamiflu, Relenza)
- Yes, antibiotics (penicilline, abbocitin, surlid, bioclavid or similar)
- Yes, other
- Don’t know / Don’t recall

[]

Did you change your daily habits while you had symptoms?

[Only answer this if the following is true: Answer 'Yes' to question '5 [DCS5]' (Have you felt ill since February 1st 2020?]

Choose one of the answers below:

- ○No
- ○Yes, but I have not been on sick leave from my job/school
- ○Yes, I have been on sick leave from my job/school

[]

What do you think caused your symptoms?

[Only answer this if the following is true: Answer 'Yes' to question '5 [DCS5]' (Have you felt ill since February 1st 2020?]

Choose one of the answers below:

- ○COVID-19
- ○Influenza or a flue-like disease
- ○A cold
- ○Allergies
- ○Asthma
- ○Gastro-intestinal infection
- ○Other
- ○Don't know

Behavior

[]Did you take any of the following precautions due to the risk of contracting corona virus? (Choose all relevant answers)

Choose one or more answers:

- ☐Wash hands more frequently
- ☐Cough or sneeze into your elbow
- ☐Use a handkerchief
- ☐wear a disposable mask
- ☐Avoid hand shakes
- ☐Stopped greetings by hugging or kissing on the cheek
- ☐Limit use of public transportation
- ☐Avoid large gatherings
- ☐Stay at home
- ☐Work from at home more often
- ☐Avoid travelling abroad or outside of your county
- ☐None of the above

For women

[]

Only answer this if the following is true:
Answer Woman' to question '1 [MK]' (Are you a man or a woman?)

Choose one answer for each element:

	Yes	No	Don’t know
**Are you pregnant or have you given birth within the past six months?**	○	○	○

Chronic disease

[]For each of the following conditions please state whether you have suffered from this now or previously.

Choose one answer for each element:

	No, I have never had that	Yes, I have that now	Yes, I had that in the past
**Asthma?**	○	○	○
**Allergy (other than asthma)?**	○	○	○
**Diabetes mellitus?**	○	○	○
**Hyperthyroidism?**	○	○	○
**Hypothyroidism**	○	○	○
**high blood pressure?**	○	○	○
**heart attack?**	○	○	○
**Chest pain (angina pectoris)?**	○	○	○
**stroke**	○	○	○

	No, I have never had that	Yes, I have that now	Yes, I had that in the past
**chronic bronchitis or smokers lungs (emphysema, COPD)?**	○	○	○
**osteoarthritis?**	○	○	○
**rheumatoid arthritis?**	○	○	○
**osteoporosis?**	○	○	○
**cancer?**	○	○	○
**other?**	○	○	○

Extra question

[]The following regards height, weight and smoking.

What is your current weight?

Choose one of the answers below:

- ○Below 50 kg
- ○50 kg
- ○51 kg
- ○52 kg
- ○53 kg
- ○54 kg
- ○55 kg
- ○56 kg
- ○57 kg
- ○58 kg
- ○59 kg
- ○60 kg
- ○61 kg
- ○62 kg
- ○63 kg
- ○64 kg
- ○65 kg
- ○66 kg
- ○67 kg
- ○68 kg
- ○69 kg
- ○70 kg
- ○71 kg

- ○72 kg
- ○73 kg
- ○74 kg
- ○75 kg
- ○76 kg
- ○77 kg
- ○78 kg
- ○79 kg
- ○80 kg
- ○81 kg
- ○82 kg
- ○83 kg
- ○84 kg
- ○85 kg
- ○86 kg
- ○87 kg
- ○88 kg
- ○89 kg
- ○90 kg
- ○91 kg
- ○92 kg
- ○93 kg
- ○94 kg
- ○95 kg
- ○96 kg
- ○97 kg
- ○98 kg
- ○99 kg
- ○100 kg
- ○101 kg
- ○102 kg
- ○103 kg
- ○104 kg
- ○105 kg
- ○106 kg
- ○107 kg
- ○108 kg
- ○109 kg
- ○110 kg
- ○111 kg
- ○112 kg
- ○113 kg
- ○114 kg
- ○115 kg
- ○116 kg
- ○117 kg
- ○118 kg
- ○119 kg
- ○120 kg
- ○121 kg
- ○122 kg
- ○123 kg
- ○124 kg

- 125 kg
- 126 kg
- 127 kg
- 128 kg
- 129 kg
- 130 kg
- 131 kg
- 132 kg
- 133 kg
- 134 kg
- 135 kg
- 136 kg
- 137 kg
- 138 kg
- 139 kg
- 140 kg
- 141 kg
- 142 kg
- 143 kg
- 144 kg
- 145 kg
- 146 kg
- 147 kg
- 148 kg
- 149 kg
- 150 kg
- Above 150 kg

[]What is your height?

Choose one of the answers below:

- 140 cm
- 141 cm
- 142 cm
- 143 cm
- 144 cm
- 145 cm
- 146 cm
- 147 cm
- 148 cm
- 149 cm
- 150 cm
- 151 cm
- 152 cm
- 153 cm
- 154 cm
- 155 cm
- 156 cm
- 157 cm
- 158 cm
- 159 cm

- 160 cm
- 161 cm
- 162 cm
- 163 cm
- 164 cm
- 165 cm
- 166 cm
- 167 cm
- 168 cm
- 169 cm
- 170 cm
- 171 cm
- 172 cm
- 173 cm
- 174 cm
- 175 cm
- 176 cm
- 177 cm
- 178 cm
- 179 cm
- 180 cm
- 181 cm
- 182 cm
- 183 cm
- 184 cm
- 185 cm
- 186 cm
- 187 cm
- 188 cm
- 189 cm
- 190 cm
- 191 cm
- 192 cm
- 193 cm
- 194 cm
- 195 cm
- 196 cm
- 197 cm
- 198 cm
- 199 cm
- 200 cm
- 201 cm
- 202 cm
- 203 cm
- 204 cm
- 205 cm
- 206 cm
- 207 cm
- 208 cm
- 209 cm
- 210 cm
- 211 cm
- 212 cm

- ◯213 cm
- ◯214 cm
- ◯215 cm
- ◯216 cm
- ◯217 cm
- ◯218 cm
- ◯219 cm
- ◯220 cm
- ◯221 cm
- ◯222 cm
- ◯223 cm
- ◯224 cm
- ◯225 cm
- ◯226 cm
- ◯227 cm
- ◯228 cm
- ◯229 cm
- ◯230 cm

[]Do you smoke? (except e-cigarettes)

Choose one of the answers below:

- ◯No
- ◯Yes, sometimes
- ◯Yes, daily, less than 10 times a day
- ◯Yes, daily, 10 or more times a day
- ◯Do not wish to answer

[]Do you use E-cigarettes (electronic cigarettes)?

Choose one of the answers below:

- ◯No
- ◯Yes, sometimes
- ◯Yes, daily, less than 10 times a day
- ◯Yes, daily, 10 or more times a day
- ◯Do not wish to answer

[]Did you drink alcohol at any time point during <u>the past 12 months?</u>

Choose one of the answers below:

- ◯Yes
- ◯No
- ◯Don’t know / Do not wish to answer

[]How many days a week do you drink alcohol?

Only answer this if the following is true:
Answer 'Yes' to question '27 [D4EF]' Have you had alcohol at any time point during the past 12 months?)

Choose one of the answers below:

- 0-1 day
- 2 days
- 3 days
- 4 days
- 5 days
- 6 days
- 7 days

[]

How many units of alcohol do you typically drink <u>a week</u>?

1 unit = 1 regular beer or 1 glass of red-/white wine or 1 alcohol soda or alcohol cider or 1 glass of liqueur or 1 drink/cocktail or 1 snaps/shot

Only answer this if the following is true:
Answer 'Yes' to question '27 [D4EF]' (Have you had alcohol at any time point during the past 12 months?)

Choose one of the answers below:

- 0-1
- 2
- 3
- 4
- 5
- 6
- 7
- 8
- 9
- 10
- 11
- 12
- 13
- 14
- 15
- 16
- 17

- ○18
- ○19
- ○20
- ○21
- ○22
- ○23
- ○24
- ○25
- ○26
- ○27
- ○28
- ○29
- ○30
- ○31
- ○32
- ○33
- ○34
- ○35
- ○36
- ○37
- ○38
- ○39
- ○40
- ○41
- ○42
- ○>42

Work and education

The following questions are related to your education and line of work

[]

What is your highest completed education?

Choose one of the answers below:

- ○I have no formal education
- ○Primary school
- ○Secondary education
- ○Vocational education or short-/medium-cycle higher education
- ○Long-cycle higher education
- ○Don’t know

[]

What was your primary line of work before the corona pandemic?

Choose one of the answers below:

- ○Full time job
- ○Part time job
- ○Self-employed
- ○Student
- ○Stay-at home
- ○Unemployed
- ○Long-term sick leave or parental leave
- ○Retired
- ○Other

[]

Do you work within the health care sector?

[Only answer this if the following the answer to “What was your primary line of work before the corona pandemic” was ‘full time job’, ‘part time job’, ‘self-employed’, ‘long-term sick leave or parental leave’ or ‘other’]:

Choose one of the answers below:

- ○No
- ○Yes, but I don’t have any patient contact
- ○Yes, I work with patients
- []

Have your job situation changed after the corona pandemic?

Only answer this if the following is true:
Answer 'Self-employed' *or* 'Full-time' *or* 'Part time' *or* 'Student' to question '31 [DCA2]' What was your primary line of work before the corona pandemic?)

Choose one of the answers below:

- ○No, I work as usual before the corona pandemic
- ○Yes, I work / study at home
- ○Yes, I have been sent home from work without any tasks
- ○Yes, I have taken vaction/time off
- ○Yes, I have been fired
- ○Yes, I work, but my job situation has changed
- ○Yes, my job situation was temprarily changed but is now back to what it used to be before the corona pandemic
- ○Yes, my job situation has changed due to parental leave
- ○Yes, my job situation has changed due to illness other than COVID-19
- ○Don’t know

[]

In what zip code is your work / education placed (the place where you spent most of your studying/working hours before the corona pandemic)?

Only answer this if the following is true:
Answer 'Self-employed' *or* 'Full-time' *or* 'Part time' *or* 'Student' to question '31 [DCA2]' What was your primary line of work before the corona pandemic?)

Choose one of the answers below:

- ○1000-1473
- ○1500-1799
- ○1800-1999
- ○2000
- ○2100
- ○2200
- ○2300
- ○2400
- ○2450
- ○2500
- ○2600-2690
- ○2700-2791
- ○2800-2880
- ○2900-2990
- ○3000-3080
- ○3100-3150
- ○3200-3250
- ○3300-3390
- ○3400-3490
- ○3500-3550
- ○3600-3670
- ○3700-3790
- ○4000-4070
- ○4100-4190
- ○4200-4296
- ○4300-4390
- ○4400-4490
- ○4500-4593
- ○4600-4690
- ○4700-4793
- ○4800-4895
- ○4900-4990
- ○5000
- ○5200
- ○5210
- ○5220
- ○5230
- ○5240
- ○5250
- ○5260

- ○5270
- ○5290
- ○5300-5390
- ○5400-5492
- ○5500-5592
- ○5600-5690
- ○5700-5792
- ○5800-5892
- ○5900-5985
- ○6000-6094
- ○6100
- ○6200-6280
- ○6300-6392
- ○6400-6470
- ○6500-6580
- ○6600-6690
- ○6700
- ○6705
- ○6710
- ○6715
- ○6720-6792
- ○6800-6893
- ○6900-6990
- ○7000-7080
- ○7100-7190
- ○7200-7280
- ○7300-7362
- ○7400-7490
- ○7500-7570
- ○7600-7680
- ○7700-7770
- ○7800-7884
- ○7900-7990
- ○8000-8100
- ○8200
- ○8210
- ○8220
- ○8230
- ○8240
- ○8250
- ○8260
- ○8270
- ○8300-8382
- ○8400-8472
- ○8500-8592
- ○8600-8680
- ○8700-8783
- ○8800-8883
- ○8900-8990
- ○9000-9100
- ○9200
- ○9210
- ○9220

- 9230
- 9240
- 9260
- 9270
- 9280
- 9293
- 9300-9383
- 9400-9493
- 9500-9575
- 9600-9690
- 9700-9760
- 9800-9881
- 9900-9990
- Don’t know/Don’t recall

[]

Which of the following descriptions matches your line of work the best?

Only answer this if the following is true:
Answer 'Other' *or* 'part-time' *or* 'full-time’ *or* 'Self-employed' *or* ‘long-term sick leave or parental leave' to question '31 [DCA2]' What was your primary line of work before the corona pandemic?)

Choose one of the answers below:

- Academia or management (fx manager, doctor, nurse, engineer)
- Administration/office (fx IT-administrator, financial assistent, receptionist)
- Sales, service, leasure (fx shop assistent, waiter, fitness instructor)
- Skilled worker (fx mechanic, electrician)
- Unskilled worker (fx cleaning assistent)
- Other

[]

Within the month prior to your corona virus test, how many people were you typically in contact with during a day? (Choose the description that fits best)

Choose one of the answers below:

- More than 10 children or young people a day
- More than 10 people >65 years of age or more during the day
- Patients regardless of number
- Groups of people (more than 10 at a time)
- None of the above

Household

[]

The following questions regard your household

How many, including yourself, from the following age groups live in your household?

Choose one answer for each element:

	None	1	2	3	4	5	>5
**0-4 year olds**	○	○	○	○	○	○	○
**5-18 year olds**	○	○	○	○	○	○	○
**19-44 year olds**	○	○	○	○	○	○	○
**45-64 year olds**	○	○	○	○	○	○	○
**65+ year olds**	○	○	○	○	○	○	○

[]

Choose one answer for each element:

	None	1	2	3	4	5	>5
**How many in your household are below the age of 18 and attends school or daycare?**	○	○	○	○	○	○	○

Transportation

[]what was your primary mode of transportation before you had your first corona virus test?

Choose one of the answers below:

- ○Walking

- ○Biking
- ○Motor vehicle/scooter
- ○Car
- ○Public transportation (bus, train, subway, etc.)
- ○Other

[]Within the month prior to your first corona virus test**, how long time did you on average spend on public transportation a day (bus, train, subway)?**

Choose one of the answers below:

- ○Did not use public transportation
- ○0 - 30 minutes
- ○30 minutes -1,5 hour
- ○1,5 hour - 4 hours
- ○More than 4 hours

SF-12

[]The following questions regard your views about your health.

In general, would you say your health is?

Choose one of the answers below:

- ○Excellent
- ○Very good
- ○Good
- ○Fair
- ○Poor

[]The following questions are about activities you might do during a typical day. Does your health now limit you in these activities? If so, how much?

Choose one answer for each element:

	Yes, limited a lot	Yes, limited a little	No, not limited at all
**Moderate activities such as moving a table, pushing**	○	○	○

	Yes, limited a lot	Yes, limited a little	No, not limited at all
**a vacuum cleaner, bowling, or playing golf**			
**Climbing several flights of stairs**	○	○	○

[]During <u>the past 4 weeks</u>, have you had any of the following problems with your work or other regular daily activities <u>as a result of your</u> <u>physical health</u>?

Choose one answer for each element:

	Yes	No
**Accomplished less than you would like**	○	○
**Were limited in the kind of work or other activities**	○	○

[]

During <u>the past 4 weeks</u>, have you had any of the following problems with your work or other regular daily activities <u>as a result of any emotional problems</u> (such as feeling depressed or anxious)?

Choose one answer for each element:

	Yes	No
**Accomplished less than you would like**	○	○
**Did work or activities less carefully than usual**	○	○

[]During the <u>past 4 weeks</u>, how much <u>did pain interfere</u> with your normal work (including work outside the home and housework)?

Choose one of the answers below:

- ○Not at all
- ○A little bit
- ○Moderately
- ○Quite a bit
- ○Extremely

[]These questions are about how you have been feeling during the past 4 weeks. For each question, please give the one answer that comes closest to the way you have been feeling.

How much of the time during the past 4 weeks…

Choose one answer for each element:

	All of the time	Most of the time	A good bit of the time	Some of the time	A little of the time	None of the time
…have you felt calm & peaceful?	○	○	○	○	○	○
... Did you have a lot of energy?	○	○	○	○	○	○
... Have you felt down-hearted and blue?	○	○	○	○	○	○

[]During the past 4 weeks, how much of the time has your physical health or emotional problems interfered with your social activities (like visiting friends, relatives, etc.)?

Choose one of the answers below:

- ○All of the time
- ○Most of the time
- ○Some of the time
- ○A little of the time
- ○None of the time

The survey is completed.

Thank you very much for your help

Submit survey

Thank you for participating in this survey

Appendix 2. The Danish COVID-19 Genetic Consortium

The Danish COVID-19 Genetic Consortium is a national consortium across key players in the clinical management of COVID-19 and biomedical researchers and establish a national genetic cohort on COVID-19 patients (defined by positive PCR or antibody test) to facilitate state-of-the-art studies of host related molecular factors important for COVID-19 etiology and severity. Collaborators in the Consortium not involved as co-authors in this manuscript, but included in the Consortium, are: Lasse Boding, Jonas Bybjerg-Grauholm, Rikke Fléron, Christian Enevold, Jens Ulrik Jensen on behalf of the ProBeCovid study and Henriette Svarre Nielsen.
